# SOCIAL FRAILTY IN RECENTLY RELOCATED SEMI-INDEPENDENT OLDER ADULTS

**DOI:** 10.1093/geroni/igz038.3285

**Published:** 2019-11-08

**Authors:** Suzanne Dupuis-Blanchard, Catherine Bigonnesse, Danica Maillet, Odette Gould, Melissa Andrews, France Légaré

**Affiliations:** 1 Univeristé de Moncton, Moncton, New Brunswick, Canada; 2 Mount Allison University, Sackville, New Brunswick, Canada; 3 Dalhousie University, Halifax, Nova Scotia, Canada; 4 Université de Laval, Québec, Québec, Canada

## Abstract

Although most older adults live outside of care institutions, not all seniors choose to live in traditional family homes. Among those who relocate, some relocate too early while others are pre-frail or frail when they relocate. Social frailty – the interaction between social vulnerability and frailty – could contribute to these untimely relocations. The goal of this study was to inform the concept of social frailty by examining a population of semi-independent older adults who recently relocated to a continuum of care community. The objectives of this study were to: 1) understand the influence of the social determinants of health on the relocation process; 2) explore whether relocation increases or reduces social frailty; and 3) measure the level of post-relocation frailty in study participants. This mixed method study combined semi-structured interviews on the relocation process, the frailty identification tool PRISMA-7, and socio-demographic surveys. Twenty-nine recently relocated seniors were recruited with the assistance of a Citizens' Advisory Committee along with advertisements, presentations, information booths, and word of mouth. Qualitative descriptive thematic analysis and descriptive statistical analyses were used to examine the relationship between frailty, socio-demographic variables and relocation. Findings indicated that several social determinants contributed to frailty and that relocation into a continuum of care community could mitigate some aspects of social frailty. A conceptual framework on the influence of social frailty on relocation is discussed. More research is needed to inform the concept of social frailty and to better understand the impact of social factors on frailty.

